# The complex consequences of engineering oil leaf production in plants

**DOI:** 10.1093/plphys/kiae209

**Published:** 2024-04-09

**Authors:** Henning Kirst

**Affiliations:** Assistant Features Editor, Plant Physiology, American Society of Plant Biologists; Departamento de Genética, Campus de Excelencia Internacional Agroalimentario ceiA3, Universidad de Córdoba, 14071 Córdoba, Spain; Instituto Maimónides de Investigación Biomédica de Córdoba (IMIBIC), 14004 Córdoba, Spain

Plant-derived oleochemicals are a promising sustainable substitute for fossil fuel–based feedstocks for lubricants and polymers or as biofuels and food additives (Hill 2007). However, compared to cellulose, the plant oil content is very low and significant increases in plant oil production are needed to meet the growing global demands. Most plants accumulate relatively high amounts of oil in seeds and fruits. However, this oil accumulation occurs only during the last third of the plant lifecycle. Thus, it has been proposed to engineer the leaves of plants to constantly produce plant oils, which could considerably increase production.

Previously, transgenic tobacco plants with elevated oil content in leaves were generated using a “push-pull-protect” strategy. A push was generated by upregulating fatty acid biosynthesis using a transgenic transcription factor (Vanhercke et al. 2014). The pull was generated by conversion of the fatty acids into triacylglycerol (TAG), which were protected from degradation by packaging into lipid droplets (Vanhercke et al. 2014).

These high-oil (HO) tobacco plants produced 15% of dry weigh as TAG, but their growth was significantly impacted. Interestingly, growth could be recovered by expression of an *Arabidopsis thaliana* LEAFY COTYLEDON 2 (LEC2) transcription factor, which is a regulator of embryogenesis. In these LEC2 plants, TAG accumulated to even higher levels, about 30% of the leaf dry weight (Vanhercke et al. 2017). LEC2 is a master regulator of embryogenesis and oilseed maturation developmental programs, during which TAG accumulates as the major storage lipid (Stone et al. 2001; Angeles-Nunez and Tiessen 2011). When expressed in leaves, LEC2 leads to elevated levels of TAG in the form of cytosolic oil bodies containing oleosin, similar to how they are stored in seeds (Feeney et al. 2013, Kim et al. 2014, Santos Mendoza et al. 2005).

To investigate the metabolic fluxes of the HO and LEC2 plants, Johnson et al. (2024) used radioactive ^14^CO_2_ pulse chase labeling to reveal in planta the effects of leaf oil engineering on resource partitioning of photosynthetically fixed carbon into various metabolic products. Their findings, recently published in *Plant Physiology*, suggest that stabilizing the desired lipid end product in oleosin-storage bodies has an important role in its accumulation in leaves and also contributes to a higher photosynthetic productivity.

First, the authors compared the distribution of polar lipids (membranes) and TAG between the wild type (WT) and the HO line. While most ^14^C in the WT was associated with membrane lipids and only 1% was incorporated into TAG, this changed dramatically in the HO line, in which up to 50% less ^14^C was associated with membrane lipids compared to WT and a total of 17% was incorporated into TAG. This indicated that carbon flux was redirected toward TAG production in the HO line, as expected. However, the signal in TAG decreased over time, indicating that the formed TAG is metabolized rather than accumulating as a stable end product.

The authors examined the carbon partitioning more closely by fractioning the sample into aqueous soluble metabolites, total protein, cell wall, starch, and total lipids to identify metabolic differences between the plants. The results revealed that the oil-accumulating lines had a more rapid efflux of metabolites out of the aqueous fraction (being converted to other compounds), which they traced to a high turnover rate of organic acids in the 2 oil-producing lines. Additionally, the lipid content showed significant difference in the carbon partitioning between the 3 lines. While lipids seem to be stable in the WT and LEC2 lines, the HO line dynamically synthesized and metabolized the lipids.

Interestingly, the HO line exhibited an inverse pattern of TAG diurnal cycling compared to the other lines. In HO plants, labeled starch decreased and labeled TAG increased during the night, suggesting the breakdown of starch at night fueling TAG biosynthesis. In HO plants during the day, labeled TAG decreased along with an increase in ^14^C-starch, indicating the turnover of carbon from TAG to indirectly fuel starch production. These findings indicate that TAG turnover is high in the HO line and absent in the other lines. The results also showed that both oil-producing lines show lower photosynthetic activity, with HO about 50% and LEC2 about 80% of the WT.

The work from Johnson et al. (2024) revealed that engineering leaf metabolism to accumulate oil has a widespread impact on central metabolism, beyond fatty acid synthesis and lipid assembly. The authors present 3 hypotheses to explain the results. The first suggests that fatty acid β-oxidation and glyoxylate cycle coinciding with photorespiration in leaf peroxisomes can reduce photorespiratory flux and CO_2_ assimilation in HO lines. This could very well explain the retarded growth and low CO_2_ fixation as well as high metabolic flux in and out of TAGs. The second hypothesis proposes that TAG turnover feeds starch synthesis and impairs sink strength leading to reduced CO_2_ assimilation in the HO line. The last hypothesis is that AtLEC2 stabilizes TAG accumulation and thus prevents the TAG-starch futile cycle present in the HO line, which increases CO_2_ fixation and can at least partially restore plant growth.

In conclusion, the authors find that stability of engineered leaf oil accumulation is key to limited adverse metabolic responses in central carbon metabolism that otherwise reduce photosynthetic CO_2_ fixation and thereby growth and product yield. This becomes evident by the inefficient TAG-starch futile cycle that is ongoing in HO but is alleviated in the LEC2 line. Additional engineering might further increase productivity by fully restoring CO_2_ fixation to WT levels and thus could increase leaf oil accumulation.

**Figure 1.**
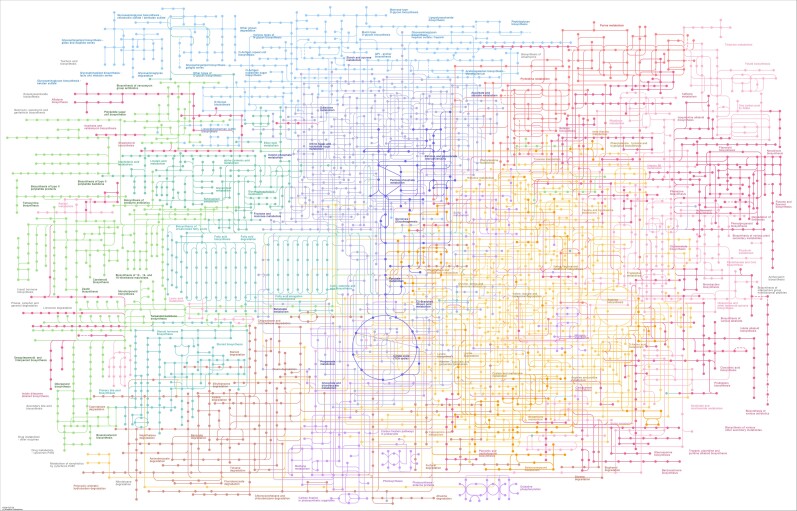
Metabolic networks within a cell illustrating their complexity. Making a change to this network can have unexpected consequences when the cell's metabolism tries to react to that change. This makes metabolic engineering challenging because controlling all possible pathways to form a product of interest is difficult and labor intensive. Figure has been created using KEGG (Kanehisa et al. 2023).

Metabolic engineering of organisms is inherently difficult. Because of the complexity of metabolic networks (Fig. 1), relatively small changes can have unexpected consequences. When intending to upregulate a certain metabolic pathway, intermediates can be taken up by off-branching pathways, which not only decreases the yields of the product of interest but also can cause toxicity or inhibit growth. This has been shown here as engineering of the tobacco plant lead to futile cycling between lipids and starch. Fortunately, studies like this one can identify where things go wrong and suggest strategies to keep production flowing.
